# Why should I prepare? a mixed method study exploring the motives of medical undergraduate students to prepare for clinical skills training sessions

**DOI:** 10.1186/1472-6920-13-27

**Published:** 2013-02-22

**Authors:** Marlien W Aalbers, Juliette Hommes, Jan-Joost Rethans, Tjaart Imbos, Arno MM Muijtjens, Maarten G M Verwijnen

**Affiliations:** 1 Skillslab, Faculty of Health, Medicine and Life Sciences, Maastricht University, Maastricht, The Netherlands

## Abstract

**Background:**

Although preparation for educational activities is considered beneficial for student learning, many students do not perform preparatory assignments. This phenomenon has received little attention in the literature although it might provide medical educators with the opportunity to enhance student learning. Therefore, we explored why students prepare or not prepare.

**Methods:**

An explorative mixed methods study was performed. In a qualitative study, 24 short group interviews with medical undergraduate students (n=209) were conducted on why they prepared for skills training sessions. In a subsequent quantitative study the resulting themes were used to construct a questionnaire. The questionnaire was presented to all undergraduate medical students at Maastricht University and 847 students completed it. Scales were constructed by a combination of exploratory factor analysis, reliability analysis, and content analysis. Between-class differences in the scale scores were investigated using ANOVA.

**Results:**

The qualitative study showed that students’ opinions on preparation are influenced by both personal factors, categorized as ‘personal learning style’, ‘attitudes and beliefs’, and ‘planning and organization’, as well as external factors, including ‘preparatory advice’, ‘pressure, consequence, and checking of preparation’, ‘teacher-related motivations’, and ‘contents and schedule of the training sessions’. The quantitative study showed that ‘the objective structured clinical examination’ and ‘facilitation of both understanding and memorizing the learning material’, were the two most motivating items. The two most demotivating aspects were ‘other students saying that preparation was not useful’ and ‘indistinct preparatory advices’. Factor analyses yielded three scales: ‘urge to learn’, ‘expected difficulties’, and ‘lack of motivation‘. Between group differences were found between the three classes on the first two scales.

**Conclusions:**

Students make an active and complex choice whether to prepare or not, based on multiple factors. Practical implications for educational practice are discussed.

## Background

Educational institutes rely on students to prepare for classes, assigning study material or independent work continually. Teachers and educational institutes try to enhance student preparation for example through providing study guides [1]. It is assumed that preparation for educational sessions is associated with improved academic achievement [2]; it is acknowledged to facilitate understanding [3] and to increase participation in class [4-6], which is known to enhance learning [7,8].

This is also acknowledged by students, who rate preparation as one of the most important factors for class participation [5]. However, we often observe that students do not always do the assigned preparatory work. An internal (unpublished) study at Maastricht University Medical School showed that 47% of first-year undergraduate students did not consistently prepare. This is not only the case for medical students: preparation rates amounted to 17% for accounting [3] and macro-economics [9] students and to approximately 30% to 50% for psychology students [10-12].

Remarkably, despite the fundamental importance of preparation and the low compliance of students, this problem has received very little attention in the literature. This was also noted by Edmunds and Brown [13], who stated that there is very little research on preparation, considering its key role in small group learning. Yet, identification of the reasons for students to prepare or not prepare is crucial, as this might help medical educators to create the most optimal learning context [14].

To our knowledge, there are no studies available that investigated why students do or do not prepare. However, preparation can be viewed as an element of self-regulated learning. It is assumed that students’ ability to regulate their own learning is essential for successful learning. This self-regulated learning behaviour is considered the product of self-generated (personal) processes and external influences [15-18]. Viewing preparation in the context of self-regulated learning behaviour infers that preparation is based on personal and external factors. Examples of personal factors are students’ motivations, personal learning styles and strategies to learn, and metacognitive judgments (are the results worth the costs?). Environmental influences, such as the nature and structure of the learning tasks, also influence students’ learning behaviour. However, it is still unclear in what way and to what extent these factors influence students’ motivation to prepare or not. Therefore the first objective of this study was to explore why students do or do not prepare for educational activities.

Furthermore, we were specifically interested to evaluate whether there are differences between classes for the factors determining students’ motivation, as it is known that advancement in university is associated with changes in learning: students in more advanced classes were more likely to read assignments in advance than beginners [11]. Moreover, students’ self-regulated learning behaviour was found to evolve as students progress through their degrees: senior students studied more and were more engaged in activities beyond the advised guidelines and relied less on others compared to first year students [19-23]. Therefore the second objective was to determine whether students’ reasons to (not) prepare differ between classes.

To study these two objectives, we conducted a mixed methods study. First we explored students’ motivations (not) to prepare by a qualitative study. Based on the results of this qualitative study, we developed a questionnaire. This questionnaire was used in the quantitative study, which is described in the second part of this article.

## General overview of the methods

We conducted a mixed method study following a complementarity sequential approach [24]. First we explored why students do and do not prepare by performing a qualitative study. This was followed by quantitative study, aimed at elucidating the importance of the reasons to (not) prepare and at evaluating if there were differences between students in different classes.

### Context

The study was conducted at Maastricht University Medical School, the Netherlands. This school offers a six-year undergraduate-entry curriculum using problem based self directed learning [25] as the central educational approach (see also [26] for more information on problem-based learning). The preclinical curriculum, comprised by the first three classes, is organised in thematic modules of six to ten weeks. Modules are offered only once a year as the modules build onto one another. As such, students belong to a class or year [1-3]. Preclinical teaching is mainly delivered in tutorial groups, complemented by lectures and a longitudinal clinical skills programme. The clinical skills programme is provided by the Skillslab and provides training in physical diagnostic, therapeutic, laboratory, and communication skills. Skills training is geared to the curriculum to ensure that the training sessions deal with subjects that are addressed in concurrent thematic modules. Skills trainings are not compulsory. Students are not assigned to training sessions, but mostly enrol themselves. As a result both group composition and teacher may vary across sessions. Preparatory advice is suggested by the faculty and is posted on Blackboard. The advice comprises information about objectives, contents, and methods of a training session (e.g. involvement of (simulated) patients, practising on each other, the need to undress), together with a list of recommended literature and (audio) visual materials. Skills performance is assessed in an annual objective structured clinical examination (OSCE). Skills-related theory is included in the regular written end-of-module tests.

### Procedures

Ethical approval was obtained from the educational management board of the Faculty. Student’s participation was completely voluntary and anonymous, and lack of participation did not have any consequences.

## Methods I qualitative study

We conducted 24 short semi-structured group interviews. Four questions were asked during these interviews to initiate the discussion (Table 1).

**Table 1:** Questions used by the moderator to guide the discussion

	
1.	What would be reasons for students to prepare for skill training sessions?
2.	What would be reasons for students not to prepare for skill training sessions?
3.	What do you think about the preparatory advice for skill training sessions?
4.	What should be changed to improve students’ preparation for skill training sessions?

The interviews were conducted during regular tutorial groups to create an environment that was completely separate from the Skillslab to minimise barriers to freely discuss preparation. Tutorial groups were randomly selected and all students that were present in the groups were asked to participate. All 209 approached students consented to participate.

Students from different classes were interviewed separately to prevent that the discussion would be dominated by higher-class students. Both literature (e.g. [27]) and the pilot interviews indicated that existing groups discussed more issues than newly established groups. Therefore we conducted the interviews in the penultimate week before the end-of-module test when tutorial groups had been together for a number of weeks.

A student moderator conducted the interviews. A student moderator was chosen instead of a staff member to minimise the chance of students withholding negative opinions. For the same reason, tutors left the room during the interviews. The moderators received extensive written and oral instructions. They tried to actively involve all students in the discussion and gave no examples of answers or summaries, since this could steer the discussion.

Interviews were audio taped and scheduled to last fifteen minutes. Two pilot sessions revealed that this design was feasible.

### Participants

Twenty-four groups were randomly selected providing approximately equal representation of students for each of the three classes, with a total of 209 students (class 1: n=75; class 2: n=61; class 3: n=72 students). Group size varied from six to twelve students. A sufficient number of group interviews was conducted, since the saturation point, i.e. the moment at which no new information came up anymore, was reached after approximately ¾ of the interviews in each class.

### Analysis

The audio recordings of the interviews were transcribed. Data were analysed by a thematic analysis approach [28,29]. Three authors and one independent reviewer independently coded the transcripts using Atlas.ti software [30]. Each reviewer defined “open” codes based on the content and assigned them to the transcripts. Next, the reviewers discussed the coding until they agreed on one coding scheme and divided codes into motivating and demotivating items. They then jointly established coordinating or “core” categories. Subsequently, they assigned codes to one or more categories if different aspects were involved. For example, ‘teacher asks questions’ belongs to the category of ‘teacher-related motivation’ but also to ‘pressure, consequences, and checking of preparation’.

## Methods II quantitative study

We designed the Preparation Questionnaire based on the results of the group interviews. Every (de)motivating item that was mentioned in more than one group interview, was translated into one question. Subsequently, the questionnaire was reviewed by four field experts and tested through a pilot study among nine preclinical students who were distributed evenly over classes 1 to 3. The comments and suggestions of both students and experts resulted in the final questionnaire, consisting of 39 items. Twenty-one items were rated on a five point Likert scale ranging from 1 ‘strongly demotivating’ to 5 ‘strongly motivating’ with 3 defined as ‘neutral’. Some items could only demotivate or motivate students to prepare; for example ‘laziness’ can only demotivate. For these questions, the five-point scale was modified into a three point Likert scale. Seven items, which could only demotivate could be rated from ‘strongly demotivating’ to ‘neutral’. Eleven items, which could only motivate, could be rated from ‘neutral’ to ‘strongly motivating’.

The 39 questions were preceded by eight general questions regarding age, gender, and preparation frequency, and perceived usefulness. The Questionnaire ended with nine specific interventions. Students were asked to indicate whether they thought that these interventions would improve their motivation to prepare.

### Participants

The questionnaire was distributed among all undergraduate students (classes 1-3) during the tutorial groups (n=847). Exchange students and foreign scholarship students were excluded. The students received both a written and an oral instruction.

### Data analysis

All analyses were performed using SPSS [31]. Descriptive statistics were reported as means and standard deviations. A p-value ≤0.05 was considered statistically significant. Cohen’s d was used as effect size for two-group comparisons, classifying effect sizes equal to .20, .50, and .80, respectively as small (negligible practical importance), medium (moderate practical importance), and large (crucial practical importance) effects, respectively [32].

#### Scale construction

The Preparation Questionnaire (39 items) was validated by constructing scales (subsets of related items), using a combination of exploratory factor analysis (Principal Components Analysis and Varimax rotation), reliability analysis (calculating Cronbach’s alpha for a scale and the change of alpha if an item is deleted from the scale), and content analysis. Inspection of the scree-plot provided the number of factors, and items with loadings <0.4, or a loading difference (between highest and next-to-highest loading) <0.1 were excluded. In a subsequent reliability and content analysis items that did not contribute to the reliability of a scale were removed. The process was iterated until a subset of items was obtained showing a consistent and interpretable factor structure, and a corresponding set of reliable scales. In the analysis Cronbach’s alpha was used as a measure of reliability (internal consistency) of a scale. After the final set of scales was constructed, corresponding scale scores (composite scores) were obtained by calculating the mean score of all items in the scale for each respondent.

#### Differing views on preparation between classes

Differences between classes were investigated by applying one-way ANOVA for each of the obtained scale scores as dependent variable, and using class (years 1, 2, and 3) as the independent variable. The Bonferroni routine was used as post-hoc test to test for significance of pair wise differences of class means.

## Results I qualitative study

We present the results according to the themes with illustrative quotations from the interviews depicted in italic.

### Preparatory advice

Students said they prepared because they wanted to know what to expect before and during the session. Special practical exercises promoted preparation, especially when the exercises were discussed. Inability to visualize a certain skill and therefore inability to understand the subject properly also resulted in less preparation. “*I can only understand it, if I’ve seen it*.” Students indicated that they would prepare more when preparatory advice is short, specific, and easy accessible.

### Contents and schedule of training session

“*You can keep up with the training session even if you have not prepared*” is a reason why students consider preparation unnecessary. Students said that the teacher often reviewed the preparatory advice at the start of the training session anyway. This rewarded students who did not prepare. It was suggested that dependence on the teacher should be reduced but at the same time some students valued review of subject matter by the teacher. “*I like it better when the preparatory advice is explained by an expert. That clarifies things for me*.”

Reasons for preparing can be summarized as increased efficiency: “*If you have prepared, everything goes faster and it’s easier to get to the point.*” Students also said that preparation depends on the type of training session: clinical reasoning sessions invited less preparation than sessions during which skills were performed.

### Planning and organization

Students preferred to do their own planning so they could choose a suitable time. The timing of a training session in relation to the concurrent module was also relevant: some students said to prepare more early during a module while others prepared more close to a test. Lack of time or planning also inhibited preparation. A related factor was that some students give less priority to skill training compared to other curricular activities. Furthermore, students said they often forget they have a training session or to prepare for it.

### Pressure, consequences, and checking of preparation

Students indicated to be more motivated when their preparation directly affected other people. For example, students said they prepared thoroughly for sessions in which they performed rectal examination on teaching associates. “*We recently learned about rectal examination. I wouldn’t like to be there without knowing where to press*”. Preparation can be promoted by pressure from the teacher, for instance by testing students’ knowledge. Conversely, a lack of stimulation by the teacher results in less preparation.

Current assessment did not stimulate preparation. “*There are only a few questions about skills in the end-of-module test…*” Lack of evaluation of professional behaviour during skill sessions and lack of repercussions demotivate: “*No one will tell you to “leave the room” or something like that if you have not prepared*.” Students thought that imposing more pressure from teachers, sanctions, or tests would improve preparation.

### Teacher-related motivations

Other teacher-related motivations included low expectations of students’ preparation by the teacher. “*You are much more motivated if you know you’ve got a training session with a teacher who really wants you to prepare than if you have a teacher who says “Well, everybody is probably unprepared”*.” Teacher characteristics played a role as well: “*I’m quite an auditory person and I know that there is a teacher that explains the material so well… That’s just fantastic… I learn more from him than from a book so I don’t read books so often since I know he will explain it so well*.”

### Personal learning style

This category is defined as “how students learn, instead of what they learn” [33]. Some students preferred studying assigned preparatory work after a training session. After not preparing for several sessions, a student was likely to develop different learning strategies and thus become habituated to not preparing. An example was ‘social loafing’ or ‘free riding’: “*There are so many people who read the information… They’ll tell me about it*.” One student suggested that this can be prevented by peer teaching.

Students also mentioned that preparation helped them to understand and learn. Having done preparatory reading helps to memorize subject matter and enables students to ask questions. Students indicated that availability of audiovisual resources greatly enhanced preparation. “*Seeing a skill performed gets through much more easily than a description of a skill*”.

Finally, only second-year students suggested to prepare because they wanted to study independently “*I like to think for myself instead of everything being predigested*”.

### Attitudes and beliefs

Many reasons for preparing were related to professionalism. “*One prepares because it’s for your own good*”. They thought that “*you should adopt a professional attitude*” and that enrolling for a training session obliges you to prepare. Moreover certain subjects were only dealt with once resulting in a strong urge to prepare: “*You won’t get a second chance*.” The perceived importance of skills for future careers was also experienced as a stimulus to prepare. Students did not only prepare because they believed it is professional to do so, but also because it makes training more enjoyable and more interesting and “*because they didn’t want to fail in the training session*.”

Some students did not prepare out of laziness, lack of interest, or when fellow students said that it was no use to prepare. Only first-year students, but not year 2 and year 3 students, mentioned that preparation was influenced by their experiences with previous training sessions.

## Results II quantitative study

The response rate was 100% (all questionnaires were returned) comprising 85.0% (n=848) of all undergraduate students. After exclusion of 39 questionnaires (4.6%) due to incomplete answers to the general questions, 809 questionnaires were included in the data analysis (n=274, n=275, and n=260 students in class 1, 2, and 3 respectively). The mean age was 20.1 years (SD 1.3) and 279 participants were men (34.5%). These distributions were similar to those of the total student population.

All students attended a skills training session at least once. The majority of the students considered preparation for a skills training valuable (mean 2.7; SD 0.5; range 1-3). Although preparation was valued useful, the mean frequency of preparation was 3.9 (SD 0.9; range 1 (almost never) to 5 (almost always)).

Strongly motivating items for preparation were: the OSCE (mean 4.4; SD 0.8), facilitation of both understanding and memorising the learning material (mean 4.3; SD 0.7 and mean 4.4; SD 0.7 respectively). The most demotivating aspects on preparation consisted of: other students saying that preparation is not useful (mean 2.2; SD 0.8), indistinct preparatory advices (mean 2.3; SD 0.8), and personal preference to study the learning material afterwards (mean 2.6; SD 0.7).

Students indicated that six out of the nine specific interventions that were proposed would improve their preparation. These interventions were: more emphasis on skills in the curriculum (indicated by 66.3% of all students), more checking whether students prepared (60.4%), more case based independent learning assignments (57.9%), more testing of skills (58.3%), more sanctions if students were not prepared (54.3%), and obligatory electronic independent assignment (54.0%).

### Aspects influencing students’ motivation to prepare

After several iterations of the factor -, reliability -, and content analyses, a set of 21 items remained that showed a consistent and interpretable three-scale structure. The scales consist of 10, 6, and 5 items, with reliabilities (Cronbach’s alpha) of 0.78, 0.58, and 0.60, respectively, and explained 36.0% of the total variance. The questions that made up the three scales are illustrated in Table 2.

**Table 2:** Overview of the 21 items affecting motivation to (not) prepare

***Scale***		***Mean***	***sd***
**Urge to learn α = 0.78**		**3.92**	**0.43**
◦	I'll know what to do	3.89	0.82
◦	I’ll know what to expect	4.02	0.77
◦	The subject treated in the training session has strong cohesion with the module	4.19	0.74
◦	I memorize the subject material better through preparation	4.35	0.70
◦	I think it’s more fun and interesting when I’m prepared	3.98	0.79
◦	It is easier to keep up with the training session	4.31	0.67
◦	Through preparation I’m able to ask questions during the training session	3.79	0.76
◦	I’d like to contribute to the training session	3.49	0.67
◦	I have the feeling that I prepare ’for myself’	4.24	0.69
◦	The training goes faster when I’m prepared	3.60	0.75
**Expected difficulties α = 0.58**		**3.75**	**0.55**
◦	The teacher has high expectations from students	3.78	1.00
◦	The subject treated in the training session is difficult	3.84	0.95
◦	During the training sessions, questions can be asked to me	4.04	0.77
◦	The skills are hard to imagine from paper	3.30	1.06
◦	The subject treated in the training is unfamiliar to me	3.75	0.97
◦	An assignment with case-based discussion in the training session	3.81	1.01
**Lack of inner drive α = 0.60**		**2.52**	**0.40**
◦	There are no consequences if I do not prepare	2.42	0.65
◦	I’m lazy	2.70	0.58
◦	I don’t feel like it	2.63	0.59
◦	Professionalism is not tested during training sessions	2.67	0.56
◦	I heard from other students that it wasn’t worth preparing for a specific training	2.22	0.80

The first scale ‘urge to learn’ comprised items linked to the urge of students to obtain knowledge. Examples of items contributing to this scale are: facilitation of memorizing the learning material, being able to ask questions, and to deliver input in the training session. The second scale was labelled ‘expected difficulties’ as the items reflected task and training related difficulties, such as new or difficult subject matter or a teacher with high expectations. The items of the third scale ‘lack of inner drive’ were related to indifference and passivity. Examples of items contributing to this scale were: no repercussions and ‘laziness’. The aspects ‘urge to learn’ and ‘expected difficulties’ motivated students to prepare (mean 3.9, SD 0.4; respectively mean 3.8, SD 0.6). The aspect ‘lack of inner drive’ demotivated students to prepare (mean 2.5; SD 0.4).

### Differing views on preparation between classes

Two out of three aspects influencing motivation to prepare, showed small to moderate differences between the three preclinical classes in their extent to (de)motivate preparation. The aspect ‘urge to learn’ motivated to prepare *less* in year three compared to years one (p<0.001, Cohens’ d=0.41) and two (p<0.001, Cohens’ d=0.43). In contrast, ‘expected difficulties’, motivated second and third year students more than year one students (p<0.001; Cohens’ d=0.44 respectively Cohens’ d=0.32). No significant between-class differences were found in students’ ‘lack of inner drive’ (p=0.087; F=2.45).

## Discussion

Although the decision whether to prepare or not is part of self-directed learning and although preparation is acknowledged to be beneficial in the students learning process, there is a lack of understanding why students do not comply with the advised preparatory work. Therefore, the current study evaluates motivations to prepare or not to prepare as expressed by students. The two parts of our mixed methods approach yielded complementary results. First, our qualitative study identified six themes and corresponding items were generated (Preparation Questionnaire). Second, in the subsequent quantitative study the questionnaire was validated and scales were constructed, providing a measurement model (scales) that enabled to investigate which aspects motivated and demotivated the undergraduate students most to (not) prepare for skills training sessions. Using the resulting scales, between class differences were found regarding the extent to which certain aspects (de)motivated students to prepare.

### Interpretation of the findings

The six themes that emerged from the qualitative study can be clearly framed in the context of self regulated learning [34]: preparation is influenced by both personal factors, consisting of ‘personal learning style’, ‘attitudes and beliefs’, as well as external factors, including categories such as ‘preparatory advice’, ‘pressure, consequence, and checking of preparation’.

In the quantitative study, three scales were identified representing motivation to (not) prepare. When looking at the contents of the scales, it becomes clear that the scales closely resemble Deci & Ryans’ self-determination theory [35]. These authors described motivation not as antagonistic behaviour such as intrinsic versus extrinsic motivation, but as a continuum of motivations based on the levels of internalization and self-determination within the individual. Our first scale, ‘urge to learn’, comprises items that resemble intrinsic motivation, e.g. *‘I think the training is more interesting or more fun’*, and identified regulation, e.g. *‘I can memorize the subject material better’*. The other two scales represent the other end of self-determination continuum composed by Deci and Ryan, mainly containing items regarding ‘amotivation’ and ‘external regulation’.

Differences between students from the three classes in their motivation to (not) prepare are in line with studies indicating that students learn how to learn: senior students study more, are more engaged in activities beyond the advised guidelines, and rely less on others compared to first year students [19-23]. In line with these studies, the quantitative study shows that second and third year students experience more motivation to prepare from ‘expected difficulties’ in the clinical skills trainings. Surprisingly, third year students relied less on intrinsic motivation. This could be due to an overall difference in an ‘urge to learn’, representing less intrinsic or identified regulation, between classes. However, it could also be explained by a reduced need to prepare because year three students are more experienced in their clinical skills and the focus of training in year three is shifted towards integration of knowledge and skills instead of learning new skills which is the major aim in year one. The results seem to indicate that students' reasons to prepare develop or change when students progress through the curriculum. However, it would require a longitudinal study to verify whether the observed between-class differences are indeed due to a development effect and not to a cohort effect. Knowing this is useful for educational institutes because they can take this into account when organizing clinical skills programmes.

Surprisingly, both the qualitative and quantitative studies show that students are merely driven by external regulation to prepare: only one reported motivation in the qualitative study to (not) prepare was purely intrinsic *“Skills trainings are more fun and interesting when I prepare”*. Moreover, students reported that their preparation would be improved by several externally regulated interventions such as more testing, more checking of preparation, and more sanctions for not preparing. Several studies demonstrated that student motivation and preparation behaviour can be strongly influenced by external factors such as upcoming tests [3,10,12]. Apparently, this also holds true for students who are used to Problem-Based Learning, a learning context in which students are supposed to study in a self-directed manner according to their own learning goals. This educational system contrasts with the adaptations that students propose to improve their motivation to prepare. This might be due to a ‘misfit’ between students’ needs and the learning context. Another explanation might be our implementation of the Problem-Based Learning curriculum for our clinical skills programme. For instance, the organization of Skillslab sessions at Maastricht medical school appears to run against the concepts of Problem-Based Learning, with assigned preparation and teachers explaining subjects in class. This is rather contrary to self-directed learning where PBL stands for. Indeed, students indicated that in order to prepare better, they would like training sessions to be more ‘student-centred’ instead of ‘teacher-centred’, suggesting that higher self-determination in trainings would improve preparation. Van den Hurk et al. [36] also stressed the importance of self-directed learning, demonstrating that low self-regulation is associated with lower and less effective preparation.

Another factor that illustrated students’ ambiguous attitude towards self-directed and teacher-directed learning was their opinion about teachers compensating for their lack of preparation by reviewing subject matter. In the majority of interviews this was very often mentioned as a negative motivation, which made students feel that preparation was a ‘waste of time’. On the other hand some students indicated during the interviews to be motivated by this factor. A similar bivalent attitude was seen in the quantitative study with some of the students experiencing reviewing the advised preparatory work as motivating, while others experienced this as demotivating. This resulted in an average neutral score. This might not only result from differences between students but also from differences between teachers. For example, the amount of time that is taken-up by the revision might be an important determinant of preparation. During the qualitative study, students clearly indicated that lengthy revision of the advised preparation material particularly decreased their motivation to prepare because this revision caused limited time to practice skills. Moreover, the way teachers review the advised preparatory assignments might influence preparation with active involvement of students being more stimulating than just summarizing the preparatory material.

That students’ motivation to prepare is rather complex was exemplified by the quantitative study, yielding three scales with only 21-items remaining to be included, and these three factors only explained 36.0% of the total variance. Another reason for the low variance explained, could be that there were other aspects, which were not included in our questionnaire although it was based on all motivations mentioned by students in the qualitative study. Finally, complexity of preparation was reflected by the discrepancy between perceived effectiveness and preparation frequency: even though students valued preparation as useful, only 26.0% of students indicated that they almost always prepare for training sessions. Students weigh the costs and benefits of preparation, they “constantly evaluate the requirements for participation in activities and choose to either engage or to find some other activity that better takes up their time” [37]. In other words, students’ meta-cognitive judgments, which are a form of personal factors in self-regulated behaviour, are very strong determinants for preparation.

### Limitations

First of all, this study is explorative in character and is an attempt to help teachers understand what drives students to prepare or not to prepare. In this mixed methods study we have shown that there are various reasons to prepare, but overall, students make an active and deliberate choice. Referring to the lack of literature on preparation, this study is one of the first to throw light on the factors that influence preparation. Therefore, we recommend replication of this study. Future research should focus on development in motivations to prepare over time, and should evaluate how proposed interventions influences students’ motivation to prepare.

Second, in the qualitative study, the unconventional short duration of the interviews may be considered a disadvantage. The interviews were short because they were conducted during regular tutorials. The random selection of the tutorials and the inclusion of a large number of students minimised the bias that can occur if students are recruited. This was necessary as the main objective of the first part of the study was to explore all possible reasons (not) to prepare. Therefore, we wanted to recruit as many different students as possible in terms of ability and interest in education. The pilot interviews were not limited in duration. These pilot studies showed that this approach was feasible and evoked active interaction between students and yielded sufficient data in 10 to 15 minutes. In the future it would be interesting to evaluate certain aspects of preparation in depth such as students’ meta-cognitive judgements and subsequent preparation through focus groups.

Third, in the quantitative part, the five and three point Likert scales were constructed for this study, because the group interviews showed that certain factors motivated some students, but demotivated others. Therefore, the scale had to include both ‘motivating’ and ‘demotivating’. Much effort has been undertaken in order to assure that this scale was clear to all students: a pilot was done with full attention to the students’ interpretation of this scale, all students received both oral and written instruction, and researchers were available to provide additional explanation if necessary.

Fourth, the study does not report to what extent teachers’ behaviour is influenced by the opinion or attitude of students with regard to preparation and especially with regard to the depth of the preparation. For example, do teachers review more of the advised preparatory materials when they notice that students didn’t prepare well enough and how do the students appreciate this?

Finally, the results reflect the opinion or attitude of students and not their actual behaviour. It would be interesting to investigate what students really do with regard to preparation and whether their behaviour is in line with their opinion.

### Implications for education

These findings offer a starting point from which learning contexts can be optimized in order to enhance students’ preparation and their learning behaviour and performance. The complexity of students’ choices to prepare and their development during their academic training impedes recommendation of one universal intervention that would enhance preparation by all students. This is supported by the finding that none of the proposed interventions could motivate more than two thirds of the students to possibly prepare more in the future for skills training sessions.

However, this diversity might also hold the solution: student preparation might be enhanced by offering students various options for preparation. On theoretical grounds, this provision of choice is assumed to enhance feelings of autonomy and in turn motivation and performance [35]. This was confirmed by a randomized study that showed that providing students with a choice of homework not only increased homework completion but also enhanced test performance [38]. Furthermore, considering the importance of self-directed learning both in terms of successful learning and preparation compliance [36], measures should be undertaken to encourage self-directed learning. This could be achieved through optimization of the educational setting and by practical measures, such as peer teaching and students testing each other’s knowledge, as suggested by the students. Moreover, students highly appreciated case-based independent work exercises and a majority of students indicated that these independent work exercises would increase their preparation. Therefore, this might be a practical design for preparation assignments.

## Conclusions

In conclusion, this study points out that students’ motivation to prepare for skills training is a very complex process that evolves over the academic years. Preparation can be viewed as a form of self-regulated behaviour, influenced by many environmental and personal factors. Future research should explore whether providing students with different choices enhances the efficacy of teaching and student performance.

## Competing interests

The authors declare that they have no competing interests.

## Authors’ contributions

The study was initiated by all authors and conducted by MWA and JH. JJR and GMV advised on the design of the study. MWA, JH, GMV conducted the analysis of the data. TjI and AMMM advised on data analysis. MWA and JH wrote the first drafts of the article, which were revised by JJR, GMV, TjI, and AMMM. All authors read and approved the final manuscript.

## Authors’ information

Marlien Aalbers (PhD) and Juliette Hommes (MD) are both MD-PhD students at Maastricht University. They completed the honours programme concerning research. From the beginning of their study they were active members of the student government, closely involved in the management, development, and evaluation of the educational programme of the medical faculty.

Jan-Joost Rethans (MD PhD) is a general practitioner by training and responsible for the simulated and standardized patients programme of the Skillslab, Maastricht University. His interests are educational research with simulated and standardized patients as a specialty.

Arno Muijtjens (PhD) works as a psychometric consultant at the Department of Educational Development and Research of the Maastricht Medical School. He specializes in methodology and psychometric data analysis in educational research, focussing on progress tests and computer-based testing.

Tjaart Imbos (PhD) is a statistician and retired member of the Department of Methodology and Statistics. He specialises in psychometrics and research methodology. His research interest is Statistics Education Research.

Maarten Verwijnen (MD), is a general physician working in medical education at Maastricht since his graduation in 1977; as one of the early staff members of the Maastricht Medical School he was involved in the development of problem based self directed learning of medical education in the Maastricht Medical School. Since 1999 he is head of the skillslab.

## Pre-publication history

The pre-publication history for this paper can be accessed here:

http://www.biomedcentral.com/1472-6920/13/27/prepub
